# Minnelide ameliorates Col4a5+/− mice by upregulating Col4a5 and alleviating endoplasmic reticulum stress

**DOI:** 10.3389/fphar.2026.1761502

**Published:** 2026-02-09

**Authors:** Bao-wei Ji, Jun-chao Liu, Xue Wang, Ye Yin, Jing-jia Zhang, Fu-jie Wen, Hong Xu, Qian Shen, Jian Yu

**Affiliations:** 1 Department of Nephrology, Children’s Hospital of Fudan University, National Children’s Medical Center, Shanghai, China; 2 Shanghai Kidney Development & Pediatric Kidney Disease Research Center, Shanghai, China; 3 National Key Laboratory of Kidney Diseases, Beijing, China; 4 Department of Traditional Chinese Medicine, Children’s Hospital of Fudan University, Shanghai, China; 5 State Key Laboratory of Genetic Engineering and National Center for International Research of Development and Disease, Collaborative Innovation Center of Genetics and Development, Institute of Developmental Biology and Molecular Medicine, School of Life Sciences, Fudan University, Shanghai, China; 6 Pediatric Internal Medicine Department I, First People’s Hospital of Kashi, Kashgar, Xinjiang, China

**Keywords:** alport syndrome, Co4a5, endoplasmic reticulum, minnelide, podocyte

## Abstract

**Background:**

Alport syndrome (AS) is a progressive hereditary nephropathy caused by mutations in collagen IV genes, notably COL4A5, leading to proteinuria and kidney failure. Current therapies using RAAS inhibitors show limited efficacy. Triptolide, the main active component of *Tripterygium wilfordii*, exhibits anti-proteinuric effects but is limited by poor solubility and toxicity. Minnelide, its water-soluble prodrug, provides a promising alternative.

**Objective:**

This study investigated the therapeutic potential and mechanisms of Minnelide in a female Col4a5 (X + X-) Alport syndrome mouse model.

**Methods:**

Mice were treated with Minnelide or vehicle for 3 months. *In vitro*, *Col4a5*+/− podocytes were treated with triptolide, with or without Col4a5 siRNA knockdown.

**Results:**

Minnelide significantly reduced proteinuria by 64.2%, improved glomerular pathology, upregulated renal Col4a5 expression, and suppressed endoplasmic reticulum (ER) stress. In podocytes, triptolide increased Col4a5 and alleviated ER stress. Col4a5 knockdown directly induced ER stress, which was reversed by triptolide treatment.

**Conclusion:**

Minnelide demonstrates potent renoprotective effects in AS by upregulating Col4a5 expression and mitigating podocyte ER stress, positioning it as a novel therapeutic candidate.

## 1 Introduction

Alport syndrome (AS) is a hereditary progressive nephropathy caused by mutations in collagen IV genes, predominantly *COL4A5*, which account for approximately 80% of cases (Savige et al., 2016; Sav et al., 2013; Hasstedt and Atkin, 1983; Gibson et al., 2021; Persson et al., 2005). These mutations impair the formation and secretion of the normal α3α4α5(IV) collagen trimer, leading to structural and functional defects in the glomerular basement membrane (GBM) (Kalluri et al., 1997; Warady et al., 2020). Consequently, patients develop proteinuria, podocyte injury, and progressive renal failure, with limited therapeutic options currently available (Savige et al., 2022; Gregorio et al., 2023).

A key pathophysiological mechanism in AS is endoplasmic reticulum (ER) stress, triggered by the accumulation of misfolded collagen IV in podocytes. This activates the unfolded protein response (UPR), ultimately promoting apoptosis and inflammatory injury (Pieri et al., 2014; Papazachariou et al., 2014; Zhang et al., 2019). Although agents such as tauroursodeoxycholic acid have shown promise in alleviating ER stress (Pieri et al., 2014; Yu et al., 2024; Vanacore, 2024), there remains an unmet need for therapies that simultaneously enhance collagen IV expression and mitigate cellular stress.

Triptolide, the main active ingredient extracted from the traditional Chinese herb *Tripterygium Wilfordii Hook F (TWHF)*, could reduce proteinuria and treat renal diseases through a series of mechanisms, such as improving the fusion of foot processes, protecting the foot cytoskeleton, and inhibiting apoptosis of podocytes (Wang et al., 2018; Liu et al., 2021; Kupchan et al., 1972; Chen et al., 2010). It has also been shown that triptolide can alleviate ERS during pulmonary fibrosis (Jiang et al., 2024). However, due to its poor solubility and bioavailability, the presence of hepatic, renal, and reproductive toxicity severely limits its clinical application (Xi et al., 2017; Cheng et al., 2021; Ren et al., 2021; Banerjee et al., 2013; isharwal et al., 2017).Researchers have synthesized a water-soluble prodrug, minnelide, which can also be metabolized into triptolide by alkaline phosphatase *in vivo* (Chugh et al., 2012; Noel et al., 2019). However, its mechanisms of action in AS—particularly regarding collagen IV restoration and ER stress modulation—remain unclear.

In this study, we investigated the therapeutic efficacy and mechanisms of Minnelide in a female *Col4a5*+/− mouse model of X-linked Alport syndrome. We demonstrate that Minnelide not only upregulates *Col4a5* expression but also alleviates ER stress, thereby attenuating proteinuria and preserving glomerular ultrastructure. Our findings position Minnelide as a novel dual-target candidate for AS therapy.

## 2 Materials and methods

### 2.1 Mice experiments

Col4a5-R471X mice (Cat. NO. NM-KI-200183) were purchased from Shanghai Model Organisms Center, Inc (Shanghai, China). A total of 20 female mice were used in this study. This included 10 wild-type (WT) mice and 10 Col4a5 (X + X-) Alport syndrome (AS) mice on a similar genetic background (C57BL/6J). All mice were aged 4–6weeks at the start of the experiment.

The mice were randomly assigned into the following four groups (n = 5 per group): WT Group: Wild-type mice treated with normal saline. WT + M Group: Wild-type mice treated with Minnelide (Shanghai SCR-Biotech Co., ltd, Shanghai, China). AS Group: Alport syndrome mice treated with normal saline. AS + M Group: Alport syndrome mice treated with Minnelide. Mice in the WT + M and AS + M groups received intraperitoneal (i.p.) injections of Minnelide at a dose of 200 μg/kg, administered twice weekly (biw). Mice in the WT and AS control groups received i.p. injections of an equal volume of normal saline on the same schedule. The treatment period lasted for 3 months.

Adriamycin nephropathy (AN) mouse model was established in 6–8 weeks C57BL/6 female mice by tail vein injection of 25 mg/kg Adriamycin (ADR) (Sigma-Aldrich, St. Louis, MO, USA, D1515). The mice were divided into three groups, the Control group, the ADR group, and the ADR + Minnelide group, with five mice in each group. Mice in the Control group were injected into the same dose of saline intraperitoneally and in the tail vein, mice in the ADR group were injected into the tail vein with ADR and intraperitoneally with an equal dose of saline, and mice in the ADR + Minnelide group were injected intraperitoneally with 200 ug/kg/d of Minnelide in addition to the tail vein injection of ADR. After 1 week, the kidneys were removed and used for RAN-seq and WB experiments. The detailed experimental protocol for mice can be found in our group’s previous articles (Ji et al., 2023a; Ji et al., 2023b; Ji et al., 2023c).

All mice were housed under standard specific pathogen-free (SPF) conditions with a 12-h light/dark cycle and had free access to food and water. All mouse experiments were approved by the Ethics Committee of Fudan University (approval number: IDM2024056a).

### 2.2 RNA-sequencing (RNA-Seq)

Total RNA was extracted from kidney tissues or podocytes using the Total RNA Extractor (Trizol) kit (Sangon Biotech, Shanghai, China) according to the manufacturer’s protocol. The renal tissue RNA-seq data analyzed in this study were generated from the same samples as described in our previous work (Ji et al., 2023a). While the sequencing data source is shared, the present analysis is novel and focused on a distinct set of genes relevant to glomerular basement membrane integrity in Alport syndrome (e.g., Col4a3, Col4a4, Col4a5, Lama4, Lama5, Lamb1, Lamb3, Hspg2). RNA integrity was evaluated by agarose gel electrophoresis, and RNA concentration and purity were measured using a NanoPhotometer spectrophotometer (IMPLEN, CA, USA) and a Qubit 2.0 Fluorometer (Invitrogen, Waltham, MA, USA).

Library preparation and sequencing: Sequencing libraries were prepared using the VAHTSTM mRNA-seq V2 Library Prep Kit for Illumina (Vazyme Biotech, Nanjing, China). Briefly, mRNA was enriched using poly-T oligo-attached magnetic beads, fragmented, and reverse-transcribed into cDNA. After adapter ligation and PCR amplification, libraries were purified and quantified using an Agilent Bioanalyzer 2100 system (Agilent Technologies, Santa Clara, CA, USA). Paired-end sequencing was performed on an Illumina NovaSeq platform (Illumina, San Diego, CA, USA) with an average depth of 40 million reads per sample.

Data processing and alignment: Raw reads were quality-assessed using FastQC (version 0.11.2) and trimmed with Trimmomatic (version 0.36). Clean reads were aligned to the mouse reference genome (GRCm38/mm10) using HISAT2 (version 2.0). Alignment quality was evaluated with RSeQC (version 2.6.1) and Qualimap (version 2.2.1).

Differential expression analysis: Gene expression levels were quantified using StringTie (version 1.3.3b) and reported as TPM (Transcripts Per Million). Differential expression analysis was performed using DESeq2 (version 1.12.4). Genes with an absolute fold change ≥2 (Figure 4), ≥1.3 (Figure 5) and a q-value (FDR) ≤ 0.001 were considered significantly differentially expressed. Functional enrichment analyses of Gene Ontology (GO) terms and Kyoto Encyclopedia of Genes and Genomes (KEGG) pathways were conducted using a hypergeometric test, with q-value (FDR) < 0.05 considered significant.

### 2.3 Western blot (WB)

Protein extraction and quantification: Proteins from kidney tissues and podocytes were extracted using RIPA lysis buffer (GB15544, Servicebio, Wuhan, China) supplemented with protease and phosphatase inhibitor cocktails. Protein concentration was determined using a BCA Protein Assay Kit (P0012, Beyotime, Shanghai, China) following the manufacturer’s instructions.

Electrophoresis and transfer: Equal amounts of protein (20 μg per lane) were separated by 10% SDS-polyacrylamide gel electrophoresis (SDS-PAGE) at 120 V for 90 min and then transferred onto NC membranes (10,600,002, Amersham, USA) using a wet transfer system at 300 mA for 60 min.

Immunoblotting: Membranes were blocked with 5% non-fat milk in Tris-buffered saline containing 0.1% Tween-20 (TBST) for 1 h at room temperature and then incubated overnight at 4 °C with the following primary antibodies: GAPDH (GB15004, Servicebio, Wuhan, China); β-actin antibodies (GB12001, Servicebio, Wuhan, China); COL4A5 (ab231957; Abcam, Cambridge, UK; GTX56030; GeneTex, Irvine, CA, USA); PERK (GB11510, Servicebio, Wuhan, China); phospho-eIF2α (#9721; Cell Signaling Technology, Danvers, MA, USA); eIF2α (GB15544, Servicebio, Wuhan, China) and BiP (GB15098, Servicebio, Wuhan, China). After washing three times with TBST (10 min each), membranes were incubated with horseradish peroxidase (HRP)-conjugated secondary antibodies (GB23301, GB23303, Servicebio, Wuhan, China) for 1 h at room temperature.

Detection and quantification: Protein bands were visualized using enhanced chemiluminescence (ECL) substrate (G2020, Servicebio, Wuhan, China) and imaged with a Tanon 5,200 Multi Chemiluminescent Imaging System (Tanon Science and Technology, Shanghai, China). Band intensities were quantified using ImageJ software (National Institutes of Health, USA). Molecular weight markers were included in all gels for reference.

### 2.4 Immunofluorescence (IF)

The paraffin sections of kidney tissue were deparaffinized and then antigenically repaired. The serum was closed to draw circles and titrated with primary antibodies for α-SMA (1:300, Servicebio, Wuhan, China, GB111364) incubated overnight at 4 °C. Secondary antibodies were added and incubated for 50 min at room temperature. The nuclei were stained with DAPI under a microscope (FV3000, Olympus) for observation.

### 2.5 Masson staining of renal tissue

Following deparaffinization, kidney sections were subjected to Masson’s trichrome staining using a commercial kit (G1006, Servicebio, Wuhan, China) according to the manufacturer’s instructions. Finally, the stained sections were mounted and imaged under an Olympus FV3000 microscope.

### 2.6 Cell culture and treatment

Primary podocytes (*Col4a5*+/−) were isolated from the kidneys of an Alport syndrome mouse model. For experiments, the cells were treated with Triptolide (TCI, Tokyo, Japan, T2899) at a concentration of 10 ng/mL for 30 min. Subsequently, the cells were harvested for Western blot analysis.

The MPC-5 was purchased from Shanghai Zhong Qiao Xin Zhou Biotechnology CO., Ltd (Shanghai, China). Cells were cultured at 37 °C in a mixture of DMEM+10% FBS+1% P/S medium. Exposure to 0.4ug/ml ADR (Sigma-Aldrich, St. Louis, MO, USA, D1515) for 24 h was used to induce podocyte injury, and 10 ng/mL Triptolide was pretreated 30 min before the exposure to treat podocyte injury.

### 2.7 Cell transfection

MPC-5 cells of four–eight generations were used, and when the cells reached 30% fusion, the serum-free medium was replaced and incubated for half an hour in serum diluted medium with Triptolide (10 ng/mL). For transfection studies, MPC-5 cells were transferred into six-well plates. The cells were then transfected using Lipofectamine 2000 transfection reagent (Invitrogen, Thermo Fisher Scientific, Waltham, MA, USA) and four ul of si-Col4a5 mixture (Shanghai GenePharma Co., Ltd., Shanghai, China). Cells were cultured in an antibiotic-free medium with Lipofectamine and anti-si-Col4a5 complex for 6 h, then the original medium was replaced and the cells were cultured for another 48 h. At the end of the incubation, proteins were extracted according to the manufacturer’s instructions and used for subsequent WB experiments.

### 2.8 Statistics

Data were analyzed using GraphPad Prism 9.0.0 (GraphPad Software, San Diego, CA, USA). All quantitative data are expressed as mean ± standard deviation (SD) unless otherwise noted in figure legends. Normality of data distribution was assessed using the Shapiro–Wilk test. Homogeneity of variances was verified with Brown–Forsythe or Bartlett’s test where appropriate.

**FIGURE 1:**
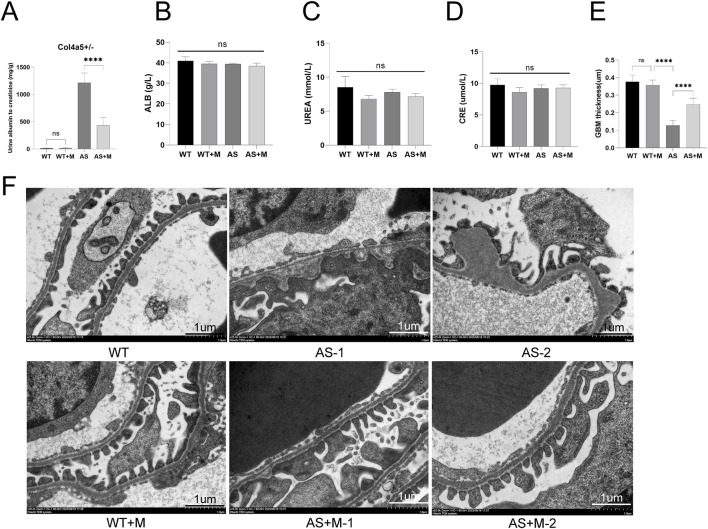
Minnelide alleviates renal function and glomerular ultrastructure in Alport syndrome mice. **(A)** Quantification of the urinary albumin-to-creatinine ratio (UACR). **(B)** Albumin (ALB) levels in each group. **(C)** Blood urea nitrogen (UREA) levels in each group. **(D)** Serum creatinine (CRE) levels in each group. Data in A-D are presented as mean ± SD; n = 5 per group. **(E)** quantitative morphometric analysis of GBM thickness across multiple capillary loops (≥10 per animal) in all experimental groups. **(F)** Representative transmission electron micrographs of glomeruli. The vehicle-treated AS mice exhibit marked glomerular basement membrane (GBM) thinning and irregularities. Minnelide treatment in AS mice ameliorates these ultrastructural defects. Scale bar: 1 μm ****p < 0.0001; NS, not significant (by two-way ANOVA with Tukey’s post-hoc test).

**FIGURE 2:**
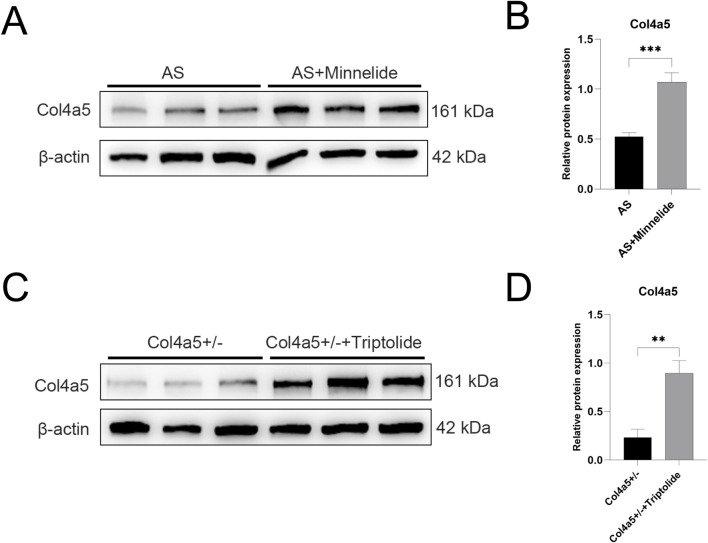
Minnelide and its active form triptolide upregulate Col4a5 protein expression *in Vivo* and *in Vitro*. **(A)** Representative Western blot images of Col4a5 in renal cortex tissues from the two experimental mouse groups treated with or without Minnelide. β-actin served as a loading control. **(B,D)** Quantification analysis of collagen IV α5 chain protein levels (refers to β-actin) in the renal tissues and podocytes of different groups. **(C)** Representative Western blot images of Col4a5 in *Col4a5*+/− primary podocytes treated with or without triptolide. β-actin served as a loading control. Data are presented as mean ± SD; n = 3 per group. **p < 0.01, ***p < 0.001; (by two-way ANOVA with Tukey’s post-hoc test for *in vivo* data).

**FIGURE 3:**
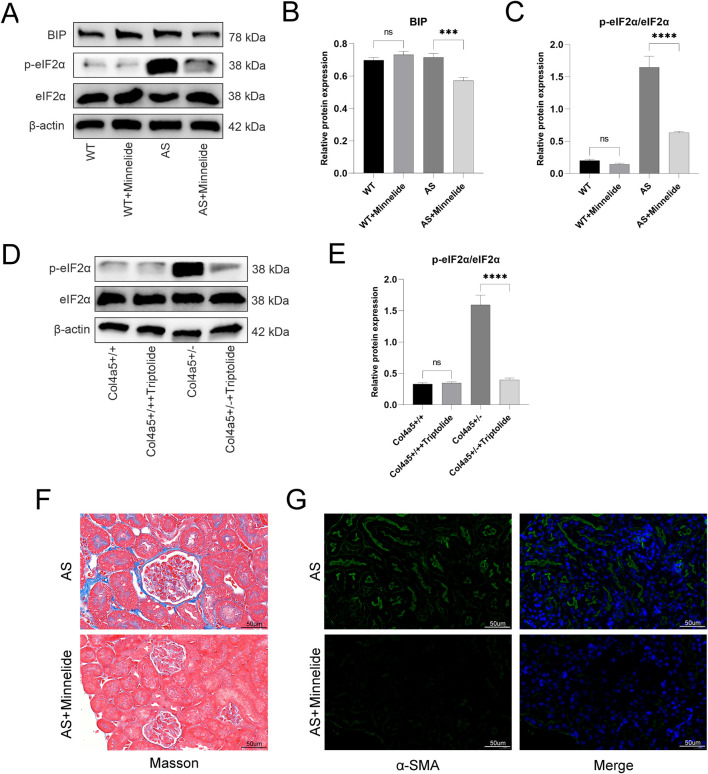
Minnelide mediates protection against fibrosis by alleviating endoplasmic reticulum stress. **(A)** Representative Western blot images of ER stress markers (BIP, p-eIF2α, total eIF2α) in renal cortex tissues from the four experimental mouse groups treated with or without Minnelide. β-actin served as a loading control. **(B,C)** Densitometric quantification of **(B)** BIP, and **(C)** the p-eIF2α/eIF2α ratio from Western blot analyses. **(D)** Representative Western blot images of ER stress markers (p-eIF2α and total eIF2α) in *Col4a5*+/− primary podocytes treated with or without triptolide. β-actin served as a loading control. **(E)** Densitometric quantification of the p-eIF2α/eIF2α ratio from Western blot analyses. **(F)** Representative images of Masson’s trichrome staining in renal cortex. Blue staining indicates collagen deposition. **(G)** Representative immunofluorescence images of α-smooth muscle actin (α-SMA) (green) in renal tissue. Nuclei are counterstained with DAPI (blue) Data are presented as mean ± SD; n = 3 per group. ***p < 0.001, ****p < 0.0001; NS, not significant (by two-way ANOVA with Tukey’s post-hoc test for *in vivo* data).

Specific statistical tests applied were as follows:

For *in vivo* studies involving two independent variables (genotype and treatment): Data in Figures 1–3 were analyzed by two-way analysis of variance (ANOVA) followed by Tukey’s post-hoc test for multiple comparisons. The urinary albumin-to-creatinine ratio (UACR) presented in Figure 1A represents a single endpoint measurement after 3 months of treatment and was analyzed accordingly.

**FIGURE 4:**
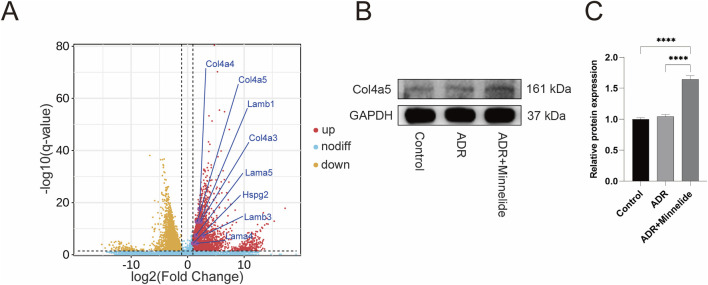
Minnelide could increase the expression of GBM-related proteins. **(A)** Volcano plots of renal tissue in AN mice (ADR + Minnelide VS. ADR). **(B)** The Western blot of COL4A5 in renal tissue of different groups. **(C)** Quantification analysis of collagen IV α5 chain protein levels (refers to GAPDH) in the renal tissues of different groups. n = 3, ****P < 0.0001 (by one-way ANOVA followed by Tukey’s post-hoc test).

**FIGURE 5:**
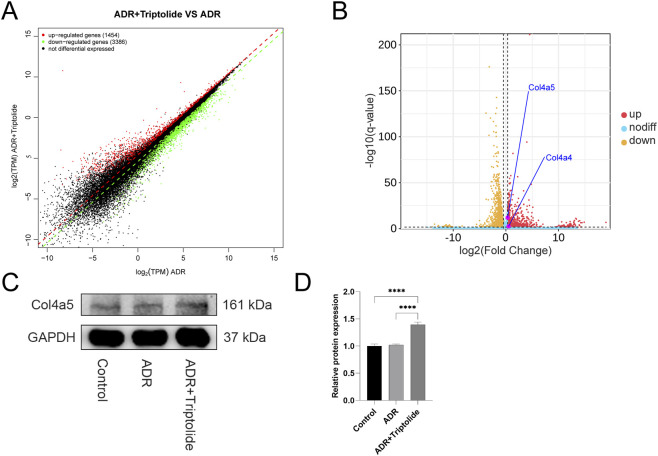
Triptolide could upregulate the expression of Col4a4/5 in podocytes. **(A,B)** Gene expression scatter plot and Volcano plots of podocytes (ADR + Triptolide VS. ADR). **(C)** The Western blot analysis of Col4a5 in different groups. **(D)** Quantification analysis of collagen IV α5 chain protein levels (refers to GAPDH) in mouse podocytes of different groups. n = 3 ****P < 0.0001 (by one-way ANOVA followed by Tukey’s post-hoc test).

**FIGURE 6:**
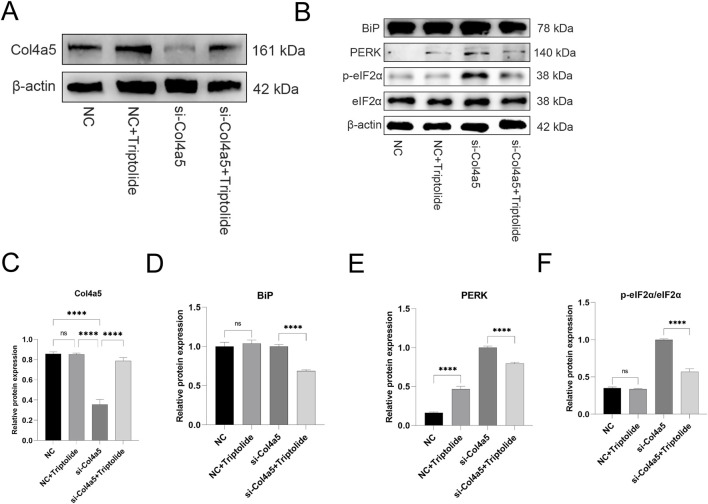
Triptolide could increase COL4A5 protein expression and alleviate endoplasmic reticulum stress in si-Col4a5 podocytes. **(A)** The Western blot analysis of Col4a5 protein levels in different groups. **(B)** The protein expression levels of BiP, PERK, p-eIF2α, and eIF2α in mouse podocytes were measured by Western blot. **(C)** Quantification analysis of Col4a5 protein levels (refers to β-actin) in mouse podocytes. **(D–F)** Quantification analysis of key ERS protein levels (refers to β-actin) in mouse podocytes of different groups. n = 3 ****P < 0.0001 (by one-way ANOVA followed by Tukey’s post-hoc test).

For *in vitro* studies with one independent variable across multiple groups: Data in Figures 4–6 were analyzed by one-way ANOVA followed by Tukey’s post-hoc test.

Statistical details for each figure panel (including the test used, number of replicates, and post-hoc correction) are provided in the respective figure legends. A p-value <0.05 was considered statistically significant.

## 3 Results

### 3.1 Minnelide ameliorates proteinuria and glomerular ultrastructural defects in alport syndrome mice

To evaluate the therapeutic efficacy of Minnelide, we treated Col4a5 (X + X-) Alport syndrome (AS) mice and their wild-type (WT) with either Minnelide (200 μg/kg, biw) or vehicle for 3 months. As anticipated, vehicle-treated AS mice developed severe proteinuria, as evidenced by a significantly elevated urinary albumin-to-creatinine ratio (UACR) compared to WT controls (Figure 1A). Notably, Minnelide treatment markedly attenuated renal albumin leakage in AS mice, resulting in an approximately 64.2% reduction in UACR (Figure 1A). Due to the absence of significant renal function decline in this Alport mouse model at 4 months of age, parameters including albumin (ALB), blood urea nitrogen (UREA), and serum creatinine (CRE) showed no notable differences between the AS and AS + M groups (Figures 1B–D).

We next assessed the glomerular ultrastructural pathology by transmission electron microscopy (TEM). Representative images revealed the characteristic lesions of Alport syndrome in vehicle-treated AS mice, including profound thinning of the glomerular basement membrane (GBM) with interspersed irregular thickening and lamellation (Figures 1E,F, AS-1 and AS-2). In stark contrast, Minnelide-treated AS mice showed a considerable preservation of GBM architecture, with a more uniform thickness and a significant reduction in pathological alterations (Figures 1E,F, AS + M-1 and AS + M-2). Together, these data demonstrate that Minnelide treatment effectively mitigates functional renal impairment and structural podocyte injury in this murine model of Alport syndrome.

### 3.2 Minnelide and its active form triptolide upregulates Col4a5 protein expression *in Vivo* and *in Vitro*

Further investigation revealed that Minnelide significantly increased the protein expression of Col4a5 in the renal tissue of Alport mice (Figures 2A,B). Consistent with this *in vivo* observation, triptolide also enhanced Col4a5 protein expression in *Col4a5*+/− podocytes *in vitro* (Figures 2C,D). These results collectively suggest that the therapeutic benefits of Minnelide, including the alleviation of proteinuria and structural improvements, may be mediated, at least in part, through the upregulation of Col4a5 expression. This restoration of the defective collagen IV α5 chain is a plausible mechanism underpinning the preservation of the glomerular basement membrane integrity and the amelioration of disease progression in Alport syndrome.

### 3.3 Minnelide mediates protection against fibrosis by alleviating endoplasmic reticulum stress

To elucidate the molecular mechanisms underlying the renoprotective effects of Minnelide, we first examined its impact on the key pathogenic defect in Alport syndrome–the deficiency of the collagen IV α5 chain (Col4a5) – and on the associated endoplasmic reticulum (ER) stress pathway in mouse kidney tissues. The expression of key ER stress markers, including the molecular chaperone BIP and the phosphorylated form of eukaryotic initiation factor 2α (p-eIF2α), was markedly elevated in the AS group, indicating activated ER stress. Minnelide treatment substantially attenuated this response, as demonstrated by the significant downregulation of both BIP and p-eIF2α (Figures 3A–C).

We next sought to determine whether the active moiety of Minnelide, triptolide, could exert a direct effect on Col4a5-deficient podocytes. We utilized a primary podocyte cell line derived from *Col4a5*+/− mice. Consistent with our *in vivo* findings, triptolide treatment effectively suppressed the baseline levels of ER stress and reduced the expression of p-eIF2α (Figures 3D,E).

Also, Minnelide treatment significantly attenuated renal fibrosis in Alport syndrome mice. Masson’s trichrome staining revealed elevated collagen deposition in AS mice, which was markedly reduced by Minnelide (Figure 3F). Consistently, immunofluorescence for α-smooth muscle actin (α-SMA) showed intensified staining in AS kidneys, indicating myofibroblast activation, and this was similarly suppressed with Minnelide therapy (Figure 3G). These results demonstrate the anti-fibrotic efficacy of Minnelide in this model.

### 3.4 Minnelide could increase the expression of GBM-related proteins

In a previous study exploring the specific mechanism by which minnelide protects mice with AN (Ji et al., 2023a), RNA-seq found that in renal tissues of AN mice after minnelide treatment, a significant increase in the expression of Col4a3/4/5, Lama4, Lama5, Lamb1, Lamb3, and Hspg2 genes, which encode type IV collagen, Laminin, and Perlecan respectively, the main constitutive proteins of GBM (Figure 4A). It was surprising to note that the expression of these genes was upregulated 2-7-fold after minnelide intervention (Table 1). Next, we conducted the current study, and it was verified that WB illustrated a significant increase in Col4a5 protein expression after minnelide intervention (Figures 4B,C).

**TABLE 1:** Detailed results of RNA-seq for GBM-related genes in renal tissue of mice.

Gene name	Mean TPM(C)	Mean TPM(B)	log2 fold change	p value	q value	Result
Col4a3	5.2559	1.0231	2.3609	5.97E-15	2.79E-13	up
Col4a4	16.8405	3.6851	2.1922	3.72E-20	4.10E-18	up
Col4a5	275.9289	83.0548	1.7322	6.36E-15	2.92E-13	up
Lama4	1.2330	0.3902	1.6600	8.84E-06	6.03E-05	up
Lama5	320.4487	111.2332	1.5265	3.55E-09	5.28E-08	up
Lamb1	133.4550	27.2425	2.2924	1.76E-15	9.14E-14	up
Lamb3	1.1308	0.1577	2.8420	4.10E-09	6.04E-08	up
Hspg2	521.9264	251.4227	1.0537	7.46E-06	5.18E-05	up

### 3.5 Triptolide could upregulate the expression of Col4a4/5 in podocytes

In renal tissues, podocytes are the sole cells that produce and secrete Col4a3/4/5 proteins. To verify whether triptolide (a metabolite of minnelide) still enhances the expression of Col4a3/4/5 genes in podocytes, we carried out an RNA-seq of podocytes.

The volcano plot shows that triptolide still enhances Col4a4/5 gene expression in podocytes, with a surprising 1.3 to 1.5-fold upregulation (Figures 5A,B; Table 2). Next, WB showed that protein expression of Col4a5 was significantly increased after treatment with triptolide (Figures 5C,D).

**TABLE 2:** Detailed results of RNA-seq for Col4a4/5 genes in podocytes.

Gene name	Mean TPM(C)	Mean TPM (B)	log2 fold change	p value	q value	Result
Col4a4	2.0072	1.3373	0.5859	0.0016	0.0068	up
Col4a5	91.1832	69.0732	0.4006	8.83E-14	2.35E-12	up

### 3.6 Triptolide could increase Col4a5 protein expression and alleviate endoplasmic reticulum stress in si-Col4a5 podocytes

To further verify whether triptolide could still upregulate Col4a5 expression under si-RNA knockdown of the Col4a5 gene, we carried out relevant experiments. The Col4a5 protein expression was upregulated after triptolide treatment (Figures 6A,C). Similarly, ERS was induced to emerge after si-Col4a5 knockdown of Col4a5, and ERS was alleviated after triptolide treatment (Figures 6B–E).

Although siRNA knockdown reduced Col4a5 mRNA levels, Triptolide treatment partially restored Col4a5 protein expression, likely through a combination of modest transcriptional upregulation and enhanced translational efficiency or protein stability under reduced ER stress.

### 3.7 Minnelide shows No hepatotoxicity or gonadotoxicity

**FIGURE 7:**
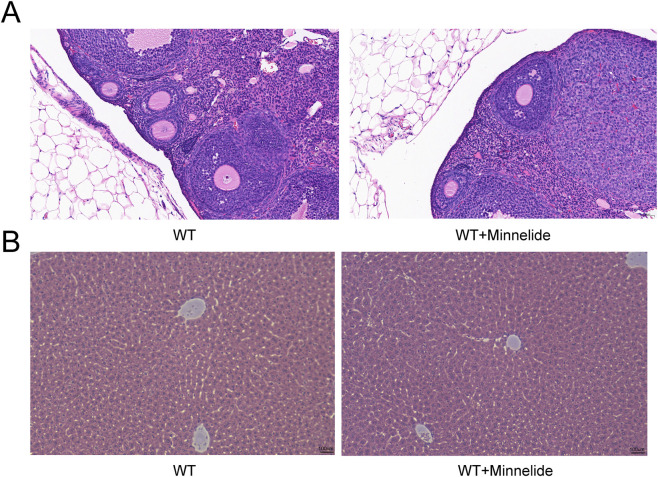
Minnelide Shows No Hepatotoxicity or Gonadotoxicity. Representative photomicrographs of **(A)** ovarian tissue and **(B)** liver tissue from wild-type (WT) and WT + Minnelide (WT + M) treated mice. Sections were stained with Hematoxylin and Eosin (H&E).

Given the documented time- and dose-dependent hepatotoxicity and reproductive toxicity associated with triptolide, we carefully evaluated the safety profile of Minnelide (200 μg/kg, biw). Histopathological examination of ovarian and liver tissues via H&E staining revealed no significant morphological alterations in the Minnelide-treated groups (WT + M) compared to their respective vehicle-treated controls (Figures 7A,B).

## 4 Discussion

Alport syndrome is a common inherited kidney disease, and it is also a major cause of inherited kidney failure, therefore, it is crucial to study the treatment of Alport syndrome (Grünfeld and Joly, 1997; Omachi and MINER, 2019). In Alport syndrome, mutations in the X-linked COL4A5 gene account for about 80% of the cases, current clinical treatment is based on RAAS inhibitors for symptoms, and there is a lack of effective therapeutic approaches (Sav et al., 2013). Moreover, gene therapy could increase the secretion of type IV collagen by podocytes, which could remodel GBM and thus treat the disease (Lin et al., 2014). Previous studies have also shown that UPR and subsequent ERS are important causes of renal injury and podocyte damage in Alport syndrome, and relieving ERS could reduce renal injury and limit disease progression (Yu et al., 2024). This study demonstrates that the water-soluble triptolide prodrug, Minnelide, confers significant renoprotection in a Col4a5 (X + X-) murine model of Alport syndrome. Our principal finding is that a 3-month treatment with Minnelide markedly attenuated disease progression, as evidenced by a pronounced 64.2% reduction in the urinary albumin-to-creatinine ratio and a notable amelioration of the characteristic glomerular basement membrane (GBM) ultrastructural defects, including thinning and irregular thickening. Mechanistically, we uncovered that the therapeutic benefits are likely mediated through a dual pathway: the upregulation of the defective Col4a5 protein and the concomitant alleviation of ERS in podocytes, both *in vivo* and *in vitro*.

The GBM is an extracellular matrix component of the glomerular filtration barrier located between podocytes and endothelial cells (Miner, 2012). It is composed mainly of collagen α3α4α5(IV) and laminin-521 (α5β2γ1). Alport syndrome is caused by mutations in any of the three GBM collagen IV chain genes (COL4A3, COL4A4, or COL4A5) (Kashtan, 1997). In a mouse model of Alport syndrome, administration of podocyte-secreted α3, α4, and α5(IV) heterotrimers to a preformed, abnormal Alport GBM could effectively restore missing collagen IV, slow the progression of renal disease, and extend life span (Lin et al., 2014). This proof-of-principle study demonstrates the plasticity of the mature GBM as well as the feasibility of a therapeutic approach to normalize the GBM to improve renal function. In the present study, we found that minnelide or triptolide could increase the expression of GBM-related proteins, especially the expression of Col4a5 protein, which could provide a theoretical basis for the future treatment of AS caused by Col4a5 gene mutation, especially the treatment of AS caused by single gene mutation.

Podocyte injury and GBM lesions are the major pathologic changes in AS. Previous studies have demonstrated that the UPR and subsequent ERS are involved in the process of podocyte damage and kidney injury in COL4A mutant models (Pieri et al., 2014; Papazachariou et al., 2014; Zhang et al., 2019; Webb et al., 2013). It has been shown that COL4A mutations affect the survival of lens fibroblasts and skin fibroblasts, possibly through the activation of UPR-associated proteins, including the protein kinases RNA-like endoplasmic reticulum kinase and translation initiation factor eIF2α (PERK-eIF2α) (Firtina et al., 2009; Wang et al., 2020). In this study, we found that ERS was induced in mouse kidneys or podocytes of AS, WB could detect the elevation of protein BiP and p-eIF2α, and the expression of these proteins could be attenuated after the treatment of minnelide or triptolide. ERS is a dysfunction of the endoplasmic reticulum that leads to the accumulation of unfolded or misfolded proteins. Adaptive ERS helps regulate protein synthesis to maintain cellular homeostasis, whereas prolonged ERS stimulation may induce apoptosis, leading to tissue and organ dysfunction and damage (Sun et al., 2024). In this study, we found that si-Col4a5 could induce ERS, as manifested by elevated PERK, BiP, and p-eIF2α, with a decrease in the expression of these proteins after treatment with triptolide.

Hereditary kidney disease is not only a major cause of ESRD in children, but also a cause of ESRD in adults (Harambat et al., 2012). Currently, there are very limited treatment options for hereditary kidney disease, and patients usually progress to ESRD, requiring dialysis or kidney transplantation, which imposes a heavy burden on society and patients. In previous studies, herbal medicines have shown great effects in a variety of diseases (e.g., cancer and diabetes), which may also play a role in the treatment of hereditary kidney diseases. Currently, some studies have reported that compounds in Chinese herbs, such as triptolide, contribute to the inhibition of the development of renal cysts and the deterioration of Autosomal dominant polycystic kidney disease (ADPKD) (Shao et al., 2021). One research used the ADPKD mouse model, the researchers examined the number, size, and proliferation rate of the cysts. By inhibiting the early stages of cyst growth, treatment with triptolide significantly improved kidney function on postnatal day 8 (Leuenroth et al., 2008). Another small clinical study showed that patients with proteinuria ADPKD experience a rapid increase in Total kidney volume (TKV) and a rapid decline in renal function, whereas Tripterygium (Triptolide-Containing Formulation) treatment significantly reduces proteinuria, slows the rate of renal TKV growth, and slows the decrease in eGFR (Chen et al., 2014). These studies suggest that triptolide has multiple effects and has a therapeutic role in ADPKD. Similarly, AS is a common hereditary kidney disease, and our study showed its ability to increase the expression of Col4a5 protein and inhibit ERS in podocytes and kidneys. Based on this, we hypothesized that minnelide and triptolide might also have some therapeutic effects on AS.

There are many limitations to our research. First, the treatment duration was 3 months, and the mice were approximately 4 months old at the endpoint. At this early disease stage, significant elevation of serum creatinine and blood urea nitrogen, markers of overt renal functional decline, had not yet manifested. Consequently, while we observed robust effects on proteinuria and histology, the long-term impact of Minnelide on preventing end-stage kidney failure in this model remains to be determined and warrants an extended treatment period. Second, regarding toxicity, the absence of detectable adverse effects is encouraging but must be interpreted with caution. Given the known time- and dose-dependent nature of triptolide’s toxicity, a more comprehensive safety assessment involving longer treatment durations and potentially higher dosing frequencies is necessary to fully delineate the therapeutic window of Minnelide for chronic kidney disease.

In conclusion, Our findings suggest that Minnelide represents a potential therapeutic candidate for Alport syndrome, operating through a dual mechanism that targets both the primary matrix defect and a key secondary injury pathway. Future studies focused on long-term efficacy and rigorous toxicological profiling will be essential to translate these encouraging preclinical results into a viable clinical strategy.

## Data Availability

The original contributions presented in the study are publicly available. This data can be found here: NCBI, accession number PRJNA1419011 (https://www.ncbi.nlm.nih.gov/bioproject/PRJNA1419011).
