# Supplementary material for: Minnelide ameliorates Col4a5+/− mice by upregulating Col4a5 and alleviating endoplasmic reticulum stress
Source: Front Pharmacol. 2026 Feb 9;17:1761502. doi: 10.3389/fphar.2026.1761502 (PMC12926658; doi:10.3389/fphar.2026.1761502)
Supplement: Supplementary file 1 [file DataSheet1.pdf]

Figure 2

Col4a5

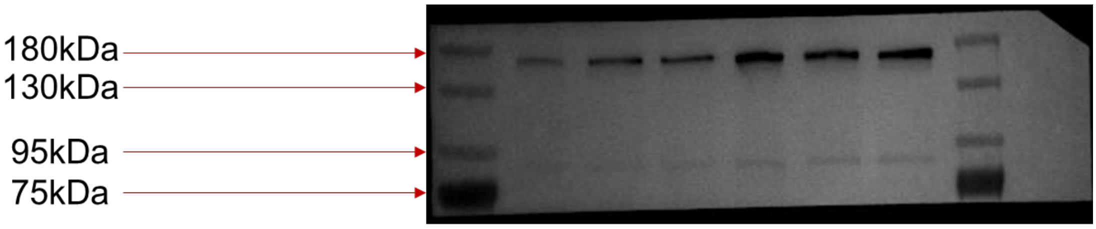

β-actin

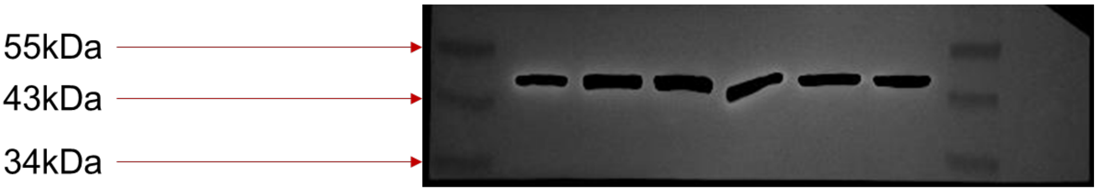

Merge

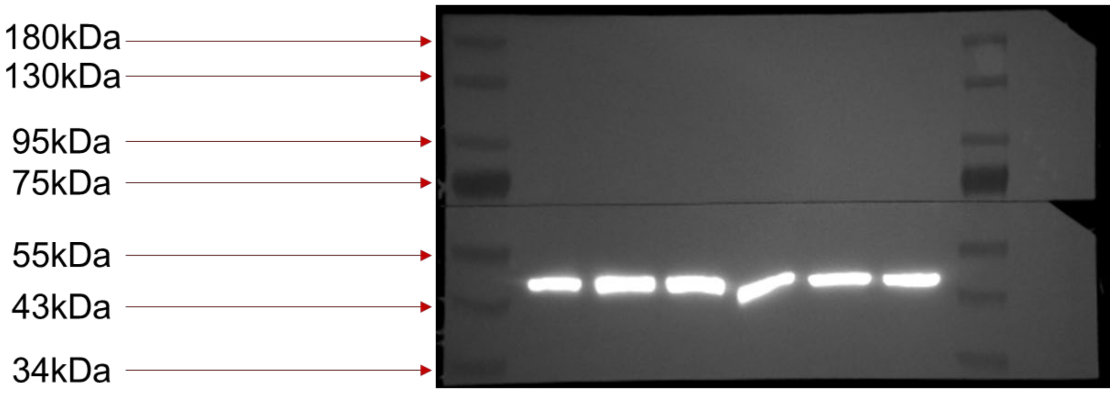

Col4a5

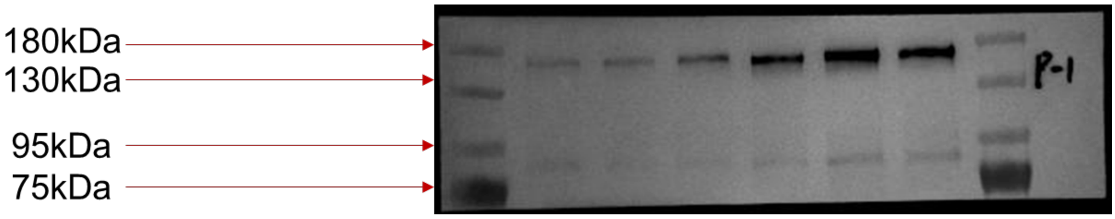

β-actin

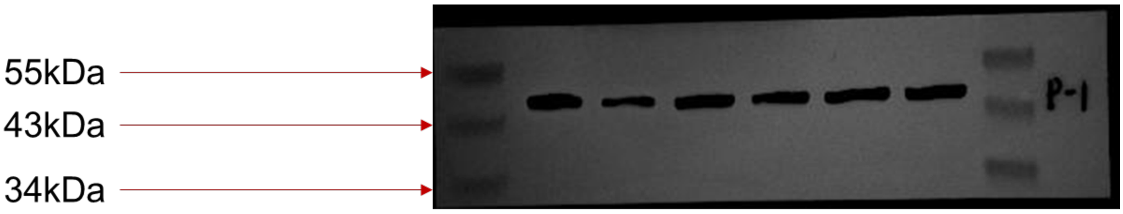

Merge

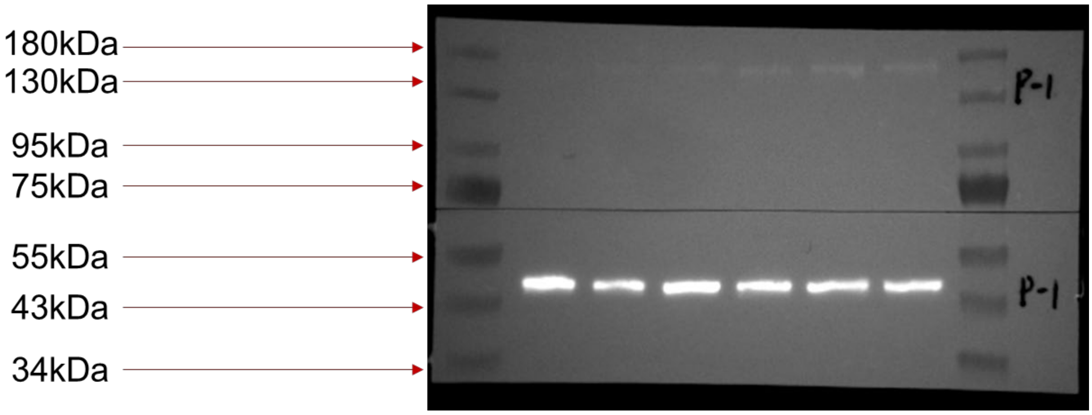
180kDa
130kDa
95kDa
75kDa
55kDa
43kDa
34kDa
P-1
P-1

Figure 3

BIP

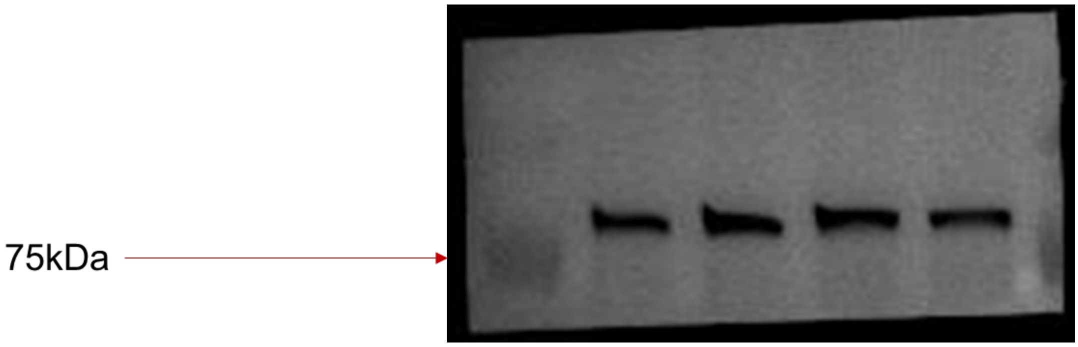

p- eIF2α

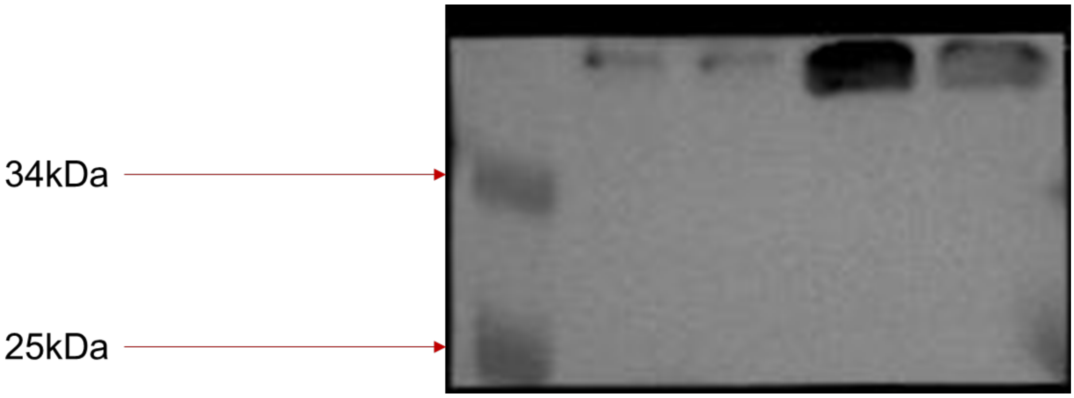

eIF2α

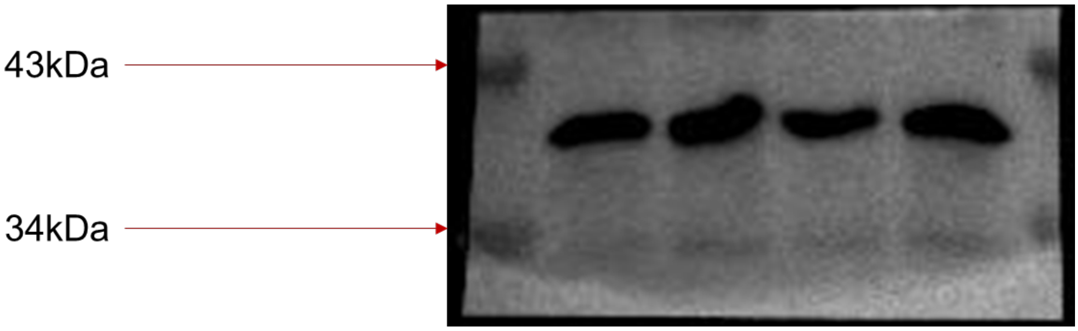

β-actin

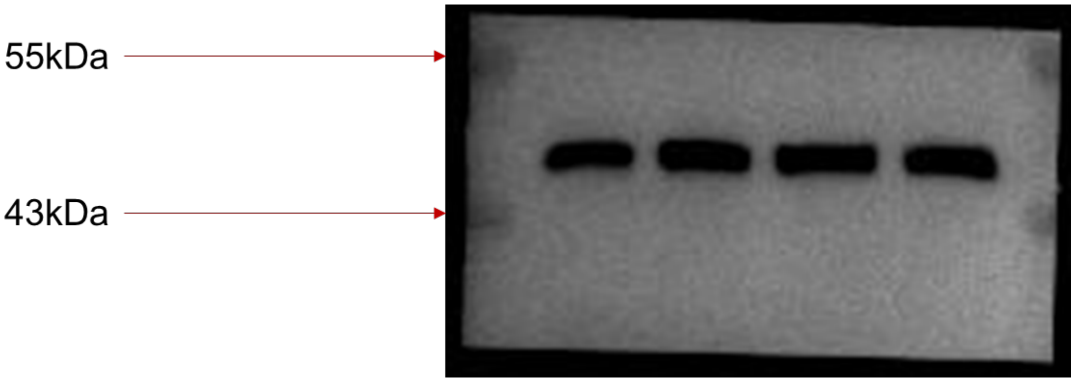

p- eIF2α

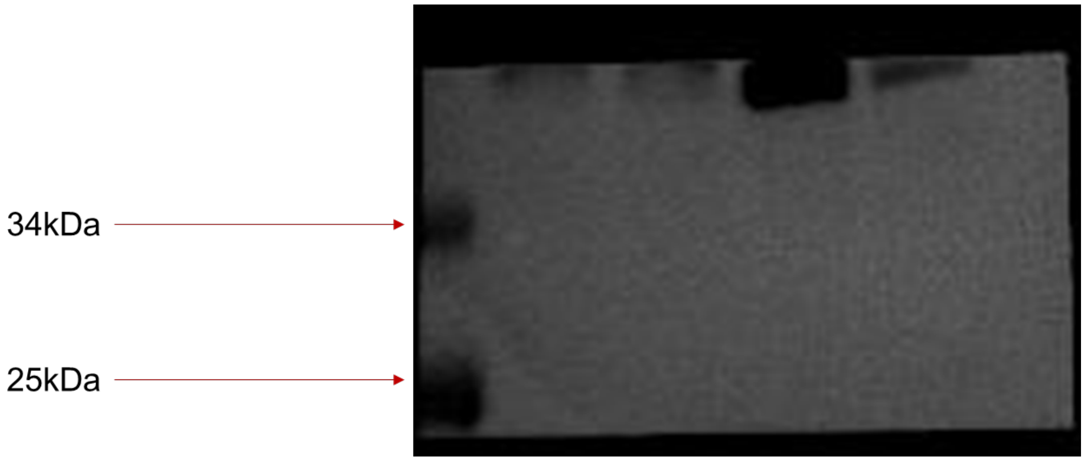

eIF2α

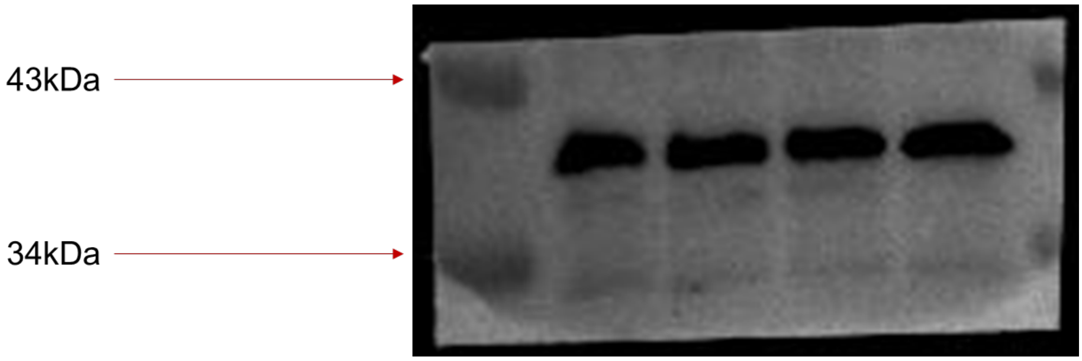

β-actin

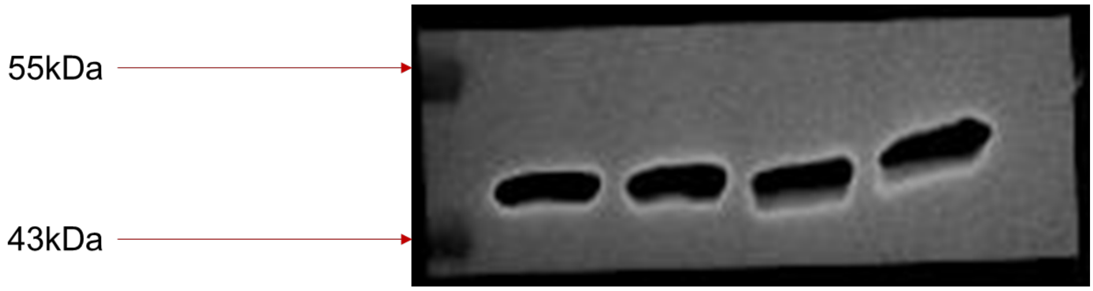

Figure 4

Col4a5

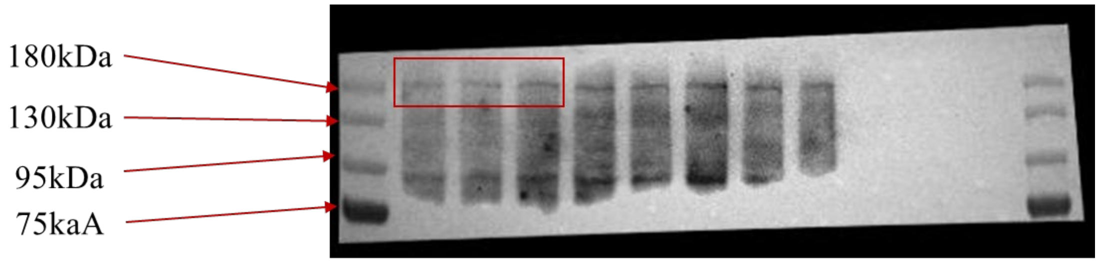

GAPDH

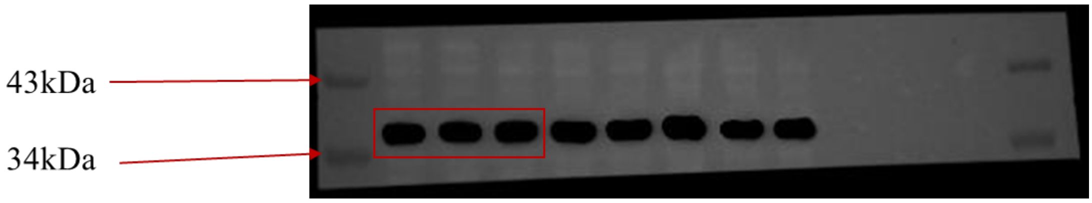

Figure 5

Col4a5

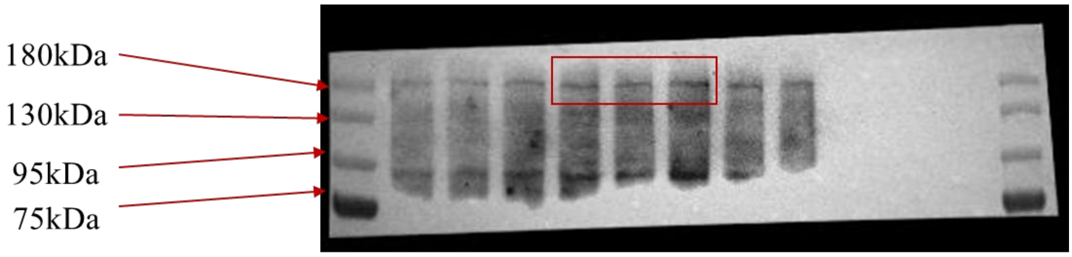

GAPDH

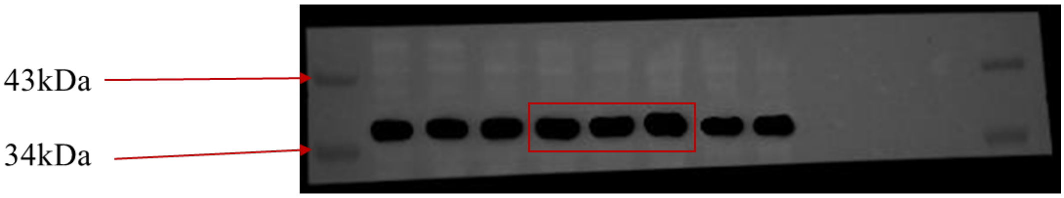

Figure 6

Col4a5

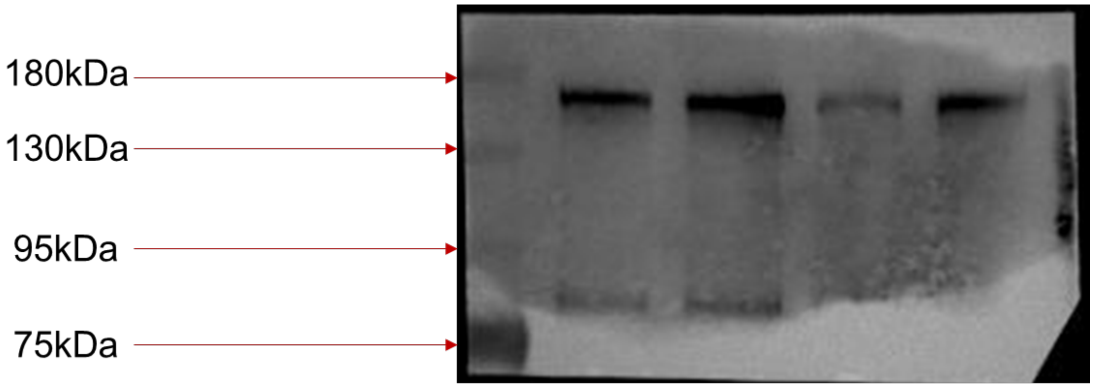

β-actin

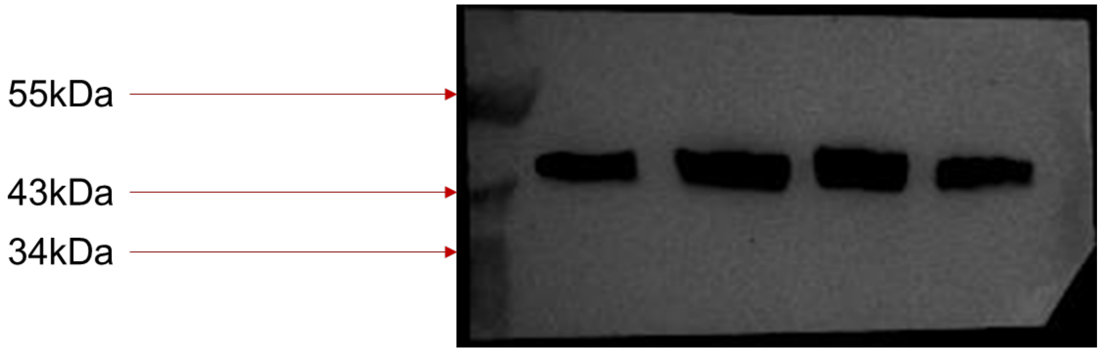

Merge

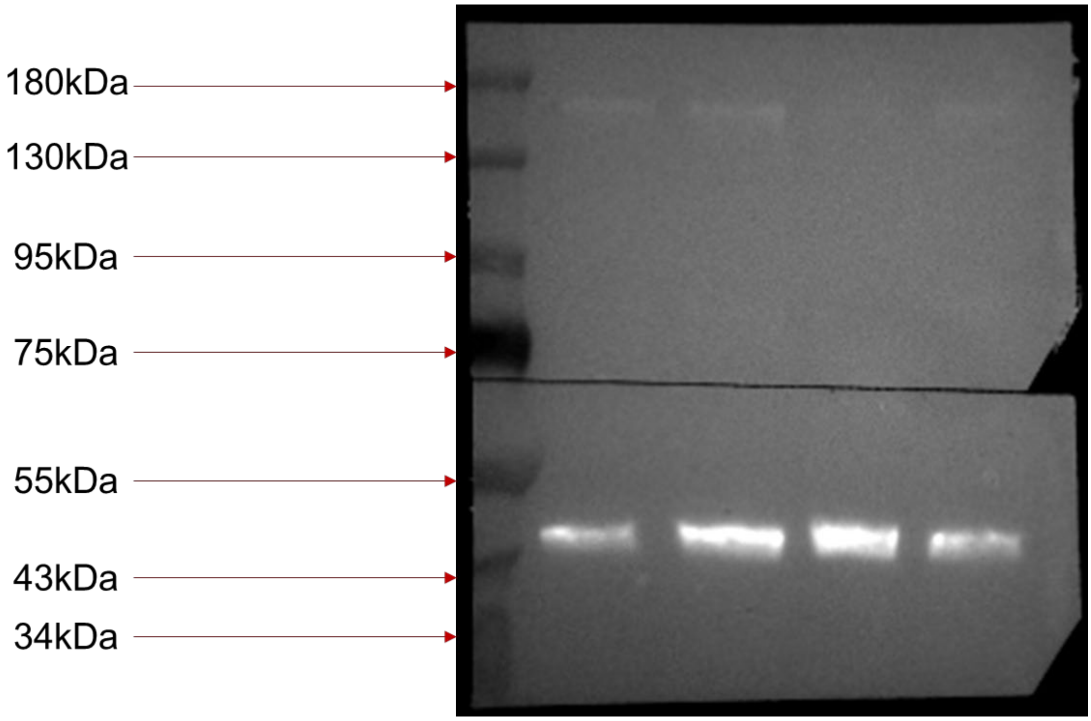

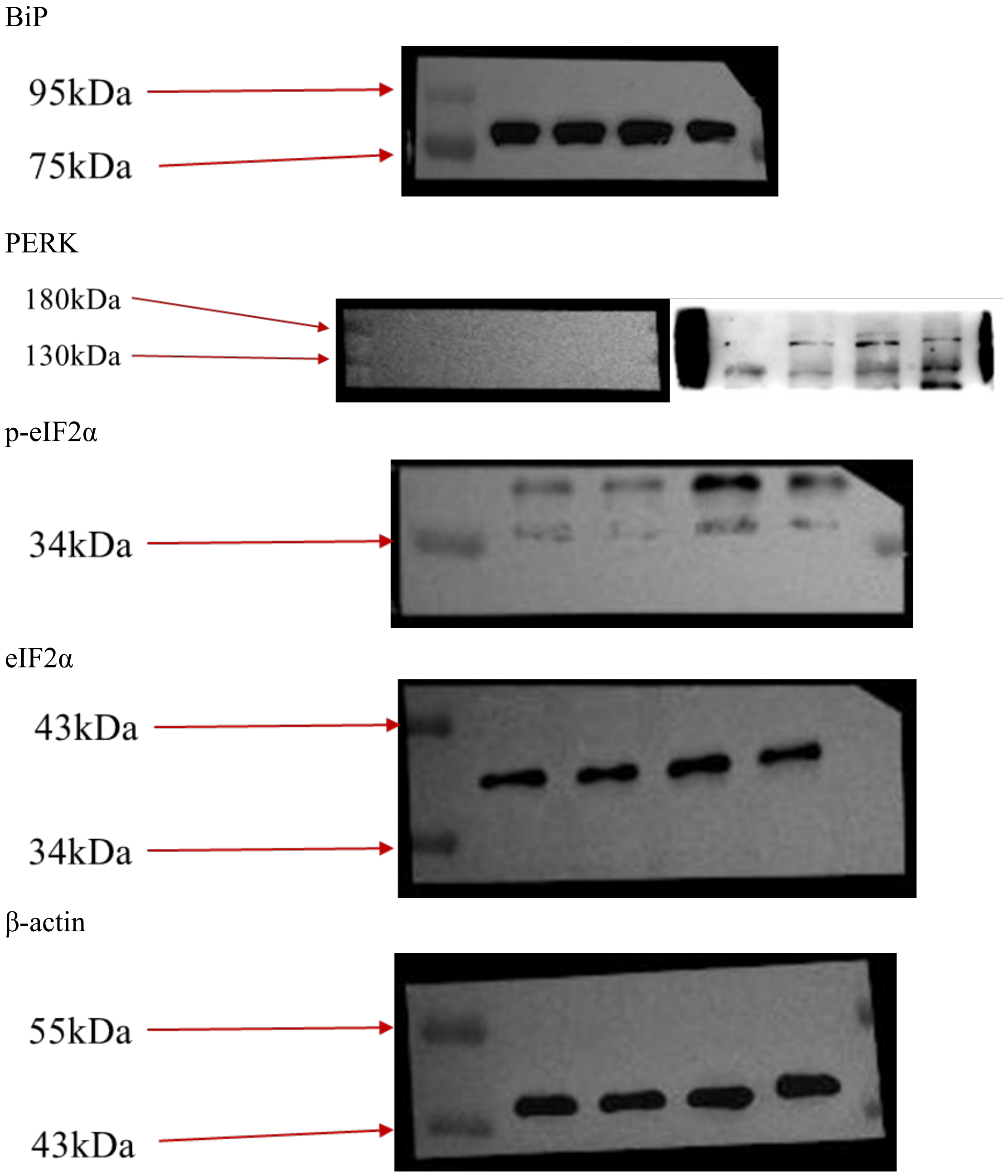
BiP
95kDa
75kDa
PERK
180kDa
130kDa
p-eIF2α
34kDa
eIF2α
43kDa
34kDa
β-actin
55kDa
43kDa
